# Sensitive and specific serological ELISA for the detection of SARS-CoV-2 infections

**DOI:** 10.1186/s12985-022-01768-4

**Published:** 2022-03-19

**Authors:** Ji Luo, Alexandra Brakel, Andor Krizsan, Tobias Ludwig, Marina Mötzing, Daniela Volke, Nicole Lakowa, Thomas Grünewald, Claudia Lehmann, Johannes Wolf, Stephan Borte, Sanja Milkovska-Stamenova, Jörg Gabert, Felix Fingas, Markus Scholz, Ralf Hoffmann

**Affiliations:** 1 Institute of Bioanalytical Chemistry, Faculty of Chemistry and Mineralogy, Universität Leipzig, Leipzig, Germany; 2 Center for Biotechnology and Biomedicine, Universität Leipzig, Leipzig, Germany; 3 Adversis Pharma GmbH, Leipzig, Germany; 4 Clinic for Infectious Diseases and Tropical Medicine, Klinikum Chemnitz gGmbH, Chemnitz, Germany; 5 Institut für Transfusionsmedizin, Universitätsklinikum Leipzig, Leipzig, Germany; 6 Department of Laboratory Medicine, Hospital St. Georg gGmbH, Leipzig, Germany; 7 ImmunoDeficiencyCenter Leipzig, Jeffrey Modell Diagnostic and Research Center for Primary Immunodeficiency Diseases, Hospital St. Georg gGmbH, Leipzig, Germany; 8 Institut für Medizinische Informatik, Statistik und Epidemiologie (IMISE), Universität Leipzig, Leipzig, Germany; 9 LIFE Forschungszentrum für Zivilisationserkrankungen, Universität Leipzig, Leipzig, Germany

**Keywords:** Coronavirus disease 2019 (COVID-19), Enzyme-linked immunosorbent assay (ELISA), Nucleocapsid protein (N-protein), Severe acute respiratory syndrome coronavirus 2 (SARS-CoV-2), Serological test

## Abstract

**Background:**

The severe acute respiratory syndrome coronavirus 2 (SARS-CoV-2) has triggered the worldwide coronavirus disease 2019 (COVID-19) pandemic. Serological assays for the detection of SARS-CoV-2 infections are important to understand the immune response in patients and to obtain epidemiological data about the number of infected people, especially to identify asymptomatic persons not aware of a past infection.

**Methods:**

We recombinantly produced SARS-CoV-2 nucleocapsid (N)-protein in *Escherichia coli*. We used the purified protein to develop an indirect enzyme-linked immunosorbent assay (ELISA) for the detection of SARS-CoV-2 specific antibodies. This ELISA method was optimized and validated with serum samples collected from 113 patients with RT-PCR-confirmed SARS-CoV-2 infections including hospitalized COVID-19 patients and 1500 control sera mostly collected before 2015 with different clinical background.

**Results:**

The optimized N-protein-ELISA provided a sensitivity of 89.7% (n = 68) for samples collected from patients with confirmed SARS-CoV-2 infections and mild to severe symptoms more than 14 days after symptom onset or a positive PCR test. The antibody levels remained low for serum samples collected in the first six days (n = 23) and increased in the second week (n = 22) post symptom onset or PCR confirmation. At this early phase, the ELISA provided a sensitivity of 39.1% and 86.4%, respectively, reflecting the time of an IgG immune response against pathogens. The assay specificity was 99.3% (n = 1500; 95% CI 0.995–0.999). Serum samples from persons with confirmed antibody titers against human immunodeficiency viruses 1/2, parvovirus B19, hepatitis A/B virus, cytomegalovirus, Epstein Barr virus, and herpes simplex virus were tested negative.

**Conclusions:**

We conclude that the N-protein-based ELISA developed here is well suited for the sensitive and specific serological detection of SARS-CoV-2 specific IgG antibodies in human serum for symptomatic infections. It may also prove useful to identify previous SARS-CoV-2 infections in vaccinated people, as all currently approved vaccines rely on the SARS-CoV-2 spike (S-) protein.

**Supplementary Information:**

The online version contains supplementary material available at 10.1186/s12985-022-01768-4.

## Introduction

In late 2019, the severe acute respiratory syndrome coronavirus 2 (SARS-CoV-2) rapidly spread from China to other countries [20, 21] initiating the worldwide coronavirus disease 2019 (COVID-19) pandemic. Until August 2021, SARS-CoV-2 infections have been confirmed in more than 243 million people including almost five million reported deaths according to the World Health Organization (WHO) (https://coronavirus.jhu.edu/map.html; accessed on October 25th, 2021).

SARS-CoV-2 is an enveloped single stranded RNA virus of the *Coronaviridae* family sharing ~ 82% of the genome with SARS-CoV-1 [3]. Similar to SARS-CoV-1, one ssRNA segment of SARS-CoV-2 encodes all four structural proteins, i.e., spike (S-), nucleocapsid (N-), membrane (M-), and envelope (E-) proteins [13, 15, 28]. The S-protein and especially its receptor-binding domain (RBD) play a crucial role in infecting host cells, as the RBD initiates the cell penetration by binding to the angiotensin-converting enzyme 2 (ACE2) receptor [28]. The N-protein participates in viral RNA package, transcription, and replication. It binds to the viral RNA forming the ribonucleoprotein core, which drives viral assembly by interacting with the other structural proteins [4, 12, 26]. Both S- and N-proteins are highly immunogenic [19, 26] allowing the development of serological SARS-CoV-2-assays [5, 16]. Serological studies on patients infected with SARS-CoV-1 showed that N-protein based ELISA provide a better sensitivity than S-protein ELISA and that anti-N-protein antibodies persist longer in serum than antibodies against other structural proteins of SARS-CoV-1 [17, 23].

Recently, several serological assays detecting antibodies specifically recognizing a SARS-CoV-2 protein have been reported [2, 18]. The sensitivity of these assays was ~ 94% for samples collected more than 14 days post symptom onset, while the specificity of these assays was ~ 95%, which may lead to many false positive tests. Although the specificity increased afterwards, based on the assay documents provided by the manufacturers, more accurate serological assays are still desired to aid in the control of the global COVID-19 pandemics. This includes also a better understanding of false positive results. Here, an indirect ELISA is reported for the serological detection of SARS-CoV-2 specific IgG antibodies directed against the N-protein expressed in *Escherichia coli*. The assay was validated with respect to sensitivity, specificity, and cut-off values using approximately 120 sera collected from persons with PCR-confirmed SARS-CoV-2 infections at different time points post infection and about 1500 control samples collected before 2015.

## Material and methods

### Reagents

Reagents were obtained from following manufacturers: Advansta Corporation (San Jose, USA): WesternBright Sirius®; Carl Roth GmbH & Co. KG (Karlsruhe, Germany): Carbenicillin disodium salt, lysozyme (≥ 45 000 FIP U/mg), ROTI®Fair Carbonate Bicarbonate Buffer pH 9.6, ROTI®Stock 10 × PBS, ROTI®Stock 10 × PBS-T, sodium chloride (≥ 99.5%), sodium dodecyl sulfate (SDS, ≥ 99.5%), sulfuric acid, Terrific-Broth- (TB-) Medium and urea (> 99.5%); GenScript Biotech BV (Leiden, Netherlands): SARS-CoV-2 Nucleocapsid protein (His-tag), SARS-CoV-2 Nucleocapsid Protein mAbs; MORPHISTO GmbH: Acetate buffer pH 5.0; Promega GmbH (Mannheim, Germany): Peroxidase-conjugated anti-human IgG antibody; Roche Deutschland Holding GmbH (Mannheim, Germany): cOmplete™ Mini EDTA-free protease inhibitor cocktail (from bovine pancreas); Seramun Diagnostika GmbH (Heidesee, Germany): TMB substrate solution; SERVA Electrophoresis GmbH (Heidelberg, Germany): Acrylamide/bis(acrylamide) (30% T, 2.67% C), BlueBlock PF 10x, Coomassie Brilliant Blue G-250, TEMED and trypsin (sequencing grade, MS approved); Sigma Aldrich Chemie GmbH (Taufkirchen, Germany): 2-mercaptoethanol (BioUltra), Antifoam Y-30 emulsion and imidazole (≥ 99.5%); Surmodics IVD, Inc. (Eden Prairie, USA): StabilZyme™ SELECT; Thermo Fisher Scientific (Waltham, Massachusetts, USA): AcroMetrix™ Inhibition Panel, SuperBlock^®^ (PBS).

### Protein expression and purification

The coding sequence of SARS-CoV-2 N-protein (NCBI accession # YP_009724397.2, downloaded from https://www.ncbi.nlm.nih.gov/protein) containing a N-terminal His-tag was codon-optimized for *Escherichia coli*, synthesized, cloned into a pET45b(+) vector (GenScript Biotech BV), and expressed in *E. coli* BL21(DE3). Briefly, an overnight culture from TB-Medium containing Carbenicillin (0.5 g/L) and Antifoam Y-30 emulsion (0.0075%, v/v) was inoculated with a single colony of *E. coli* BL21(DE3) pET45b(+)-N-protein and incubated on a horizontal shaker (180 rpm, Certomat® BS-T, B. Braun Biotech GmbH, Melsungen, Germany) at 30 °C for 17 h. Cells were harvested, resuspended in lysis buffer (PBS (337 mmol/L NaCl, 2.7 mmol/L potassium phosphate, 10 mmol/L disodium hydrogen phosphate, 2 mmol/L potassium dihydrogen phosphate) containing 50 µmol/L lysozyme and 1 tablet/10 mL protease-inhibitor-mix), and disrupted on a French® Press (Thermo Fisher Scientific, Waltham USA). For purification by immobilized metal affinity chromatography the protein concentration determined by NanoPhotometer NP80® (Implen GmbH, München, Germany) was adjusted with purification buffer (PBS, pH 7.4) to 10 g/L and imidazole added to obtain a final concentration of 25 mmol/L. The sample was loaded and proteins were eluted using a linear 25-min gradient from 25 mmol/L–0.5 mol/L imidazole in purification buffer. Fractions were collected in 1.5-min intervals and analyzed by SDS-PAGE. Fractions containing mostly the N-protein were combined, dialyzed against storage buffer (PBS), and stored at -20 °C. The purity of the N-protein was verified by SDS-PAGE. The most intense band expected to contain the N-protein was excised from the gel and the protein digested with trypsin (in-gel digest). The extracted tryptic peptides were analyzed by LC–MS. Additionally, proteins in the gel were semi-dry electroblotted onto a PVDF membrane (Bio-Rad Laboratories GmbH, Feldkirchen, Germany), blocked with BlueBlock solution at room temperature for 1 h and incubated with an anti-SARS-CoV-2 Nucleocapsid protein antibody (GenScript Biotech BV) diluted to 10,000-fold in BlueBlock solution at room temperature. After 1 h, peroxidase-conjugated anti-Human IgG-HRP (Promega GmbH, 1: 30,000 in BlueBlock) was added at room temperature. After 1 h, the bands were visualized with WesternBright™ Sirius substrate solution and the chemiluminescence was recorded on ChemiDoc MP (Bio-Rad Laboratories GmbH).

### Serum collection

Serum samples from PCR-confirmed SARS-CoV-2 positive patients were obtained from two individual donors and hospitalized patients (Krankenhaus Nordwest, Frankfurt, Germany, Klinikum Chemnitz gGmbH, Chemnitz, Germany, Institut für Transfusionsmedizin, Universitätsklinkum Leipzig, and Hospital St. Georg gGmbH, Leipzig, Germany) (Additional file 1: Tables S1–S4). These investigations represent parts of the analyzes in the COVID genetics cohort Leipzig-Chemnitz, which was approved by the Institutional Review Board of Leipzig University (reference numbers 195/20-ek and EK-allg-37/10–1). Control serum samples collected from 2009 to 2014 were enriched for older people, higher BMI, and a history of chronic obstructive pulmonary disease (COPD) to specifically test the assay specificity in populations at higher risk for severe COVID-19 conditions and with presumably a higher incidence of previous viral infections including other coronaviruses, although this information was not available (Additional file 1: Table S5). These 1500 serum samples obtained from the population-based LIFE-Adult study of the Leipzig Research Center for Civilization Disease (LIFE) [10]. All samples have been processed and stored by the team of the Leipzig Medical Biobank. Probands were collected all over the year and thus should well represent different antibody titers in response to seasonal bacterial and viral infections, e.g., influenza, coronaviruses, and rhinoviruses, as well as allergies to achieve a robust performance in epidemiological studies. Furthermore, serum samples from persons with confirmed antibody titers against human immunodeficiency viruses (HIV) 1/2, parvovirus B19, hepatitis A/B virus, cytomegalovirus, Epstein Barr virus, and herpes simplex virus were used for cross-reactivity studies (INSTAND, Düsseldorf, Germany).

### Enzyme-linked immunosorbent assay (ELISA)

Medium binding microplates (Greiner Bio-One, Frickhausen, Germany; 12xF8, PS, F-bottom) were coated with 150 ng SARS-CoV-2 N-protein per well in PBS at 4 °C overnight. All following steps were performed at room temperature. Wells were washed three times with PBS-T (300 µL) using a Hydro Flex ELISA washer (Tecan Group AG, Männedorf, Switzerland) and blocked with superblock (200 µL) for 60 min. Human serum (off-the-clot, sterile filtered; PAN-Biotech GmbH, Aidenbach, Germany) was used as negative control. The positive control consisted of an anti-SARS-CoV-2 N-protein antibody (1 g/L, GenScript Biotech BV) 100-fold diluted in the negative control human serum. Both controls and the serum samples were diluted 100-fold in sample buffer (Indical Bioscience, Leipzig, Germany) and incubated for 45 min. Wells were washed with PBS-T (300 µL) using the Hydro Flex ELISA washer before conjugate solution (100 µL/well) was added (anti-Human IgG-HRP, 1:30,000 in Stabilzyme Select). After 30 min, wells were washed three times as described above and TMB substrate solution added (100 µL/well). The reaction was stopped after 10 min by the addition of sulfuric acid (0.3 mol/L; 100 µL/well) and the absorbance recorded at 450 nm using a SUNRISE microplate reader (Tecan Group AG, Männedorf, Switzerland).

Additionally, serum samples were tested with a commercial anti-SARS-CoV-2 ELISA (IgG) kit (SARS-CoV-2 N-protein based ELISA, EUROIMMUN Medizinische Labordiagnostika AG, Lübeck, Germany) according to the manufacturer’s instruction.

To test potential interference, a positive serum sample was 128-fold diluted with plasma containing different interfering substances: hemoglobin (up to 20 g/L), bilirubin (up to 0.3 g/L), and triglycerides (up to 15 g/L).

### ELISA validation and statistical analysis

The absorbance values of serum samples were converted to sample-to-positive (S/P-) ratios using the absorbances recorded at 450 nm (OD_450_) of the positive (PC) and negative controls (NC), using the following equation:

$${\text{S/P-ratio}}\;(\%) = 100 \times ({\text{OD}}_{{{45}0}} {\text{(sample)}} - {\text{OD}}_{{{45}0}} ({\text{NC}})){\text{/(OD}}_{{{45}0}} {\text{(PC)}} - {\text{OD}}_{{{45}0}} ({\text{NC}}){)}$$

The cut-off value was determined by Receiver Operating Characteristics (ROC) to obtain the best sensitivity and specificity. ROC analysis was performed by GraphPad Prism 9.0.2 (Graph Pad Software, La Jolla, CA, USA).

### Western Blot analysis of serum samples

Control sera collected before 2015 but unexpectedly tested positive in the N-protein ELISA, were probed by an immunoblot. Commercial and in-house expressed N-proteins were separated by SDS-PAGE (0.5 µg per lane) using 12% gels. Gels were stained with brilliant Coomassie Blue G250 or semi-dry electroblotted onto a PVDF membrane (Bio-Rad Laboratories GmbH) [7]. Briefly, the membrane was blocked with BlueBlock solution at 4 °C overnight and incubated with SARS-CoV-2 positive sera, negative sera or potentially false-positive sera (1:10,000) at room temperature for one hour. After three washing steps with PBS-T for 5 min, the membrane was incubated with peroxidase-conjugated anti-human IgG-HRP (Promega GmbH, 1: 20,000 in BlueBlock) at room temperature for one hour. After washing, bands were detected with WesternBright™ Sirius substrate solution and the chemiluminescence was recorded on ChemiDoc MP (Bio-Rad Laboratories GmbH).

## Results

### Expression and purification of the full-length N-protein

**Fig. 1:**
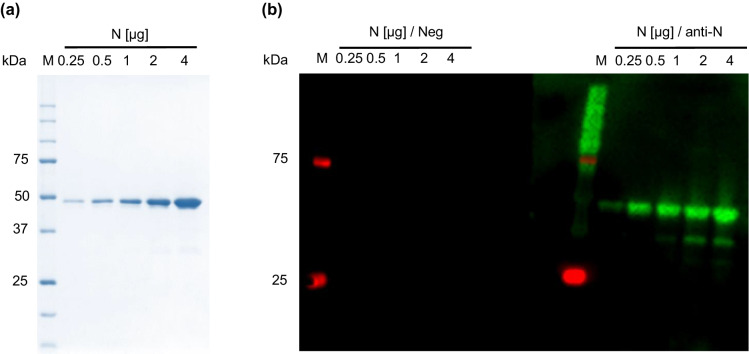
SDS-PAGE and corresponding immunoblots of the purified SARS-CoV-2 N-protein. **a** SDS-PAGE stained with Coomassie Brilliant blue (CBB) G-250 and **b** corresponding immunoblot probed with a negative serum pool (1: 10,000, Neg) and an anti-N-protein antibody (1: 50,000, anti-N) diluted in negative serum. The N-protein quantities loaded on the gel are indicated on top of each lane. M: marker proteins with the indicated molecular masses. N: N-protein

The SARS-CoV-2 N-protein was successfully expressed at high levels with N-terminal His-tag at a basal level without IPTG induction. After affinity chromatography, a single band was detected in SDS-PAGE at the expected apparent molecular weight at ~ 47 kDa (Fig. 1), which was confirmed by an immunoblot using an anti-N-protein antibody and after in-gel digestion by LC–MS. When larger quantities were loaded, a few weak bands appeared at apparent molecular weights of ~ 30 kDa, which were identified as C-terminally truncated N-proteins by LC–MS. At -20 °C the purified protein was stable in PBS containing 0.2 mol/L NaCl and 0%, 10% or 25% glycerol for the tested period of 360 days (Additional file 1: Fig. S1). When stored in the same buffers at 4 °C for 180 days, several bands appeared in SDS-PAGE below an apparent molecular weight of 47 kDa and at around 100 and 150 kDa (Additional file 1: Fig. S1).

### Nucleocapsid-protein ELISA

The indirect ELISA for anti-SARS-CoV-2 IgG antibodies was initially established using a commercial N-protein with “research grade” purity to accelerate the assay development, while the N-protein was expressed and purified in-house. First, coating of the N-protein was evaluated for carbonate buffer (pH 9.6), PBS (pH 7.4), and acetate buffer (pH 5.0) using an anti-N-protein IgG, a SARS-CoV-2 positive serum, and a negative control serum (Additional file 1: Fig. S2a). While carbonate buffer provided the highest absorbance for positive samples, the best ratio between positive and negative samples was obtained for PBS. Considering the OD_450_-values obtained for coating different protein quantities (Additional file 1: Fig. S2b) and the efforts and costs to produce pure N-protein, 150 ng of N-protein were coated per well. Using this condition, the best performance among the tested microtiter plates was observed for Maxisorp (Nunc, Roskilde, Denmark) and medium binding (Greiner) based on the ratios of the OD_450_-values measured for the anti-N-protein IgG antibody and the negative serum pool (Additional file 1: Fig. S2c). All following experiments used medium binding plates and a 30,000-fold dilution of the secondary anti-human IgG-HRP antibody in Stabilzyme Select (Additional file 1: Fig. S2d). The robustness and reproducibility of the ELISA was warranted by including positive and negative control samples to normalize the OD_450_-values percentage-wise.

The optimized ELISA using the purified in-house N-protein was probed with 68 sera collected at least 14 days after symptom onset and confirmation of a SARS-CoV-2 infection by PCR along with 1500 sera collected in Germany from 2009 to 2014 considered to be SARS-CoV-2 and SARS-CoV-1 negative. A ROC curve analysis provided a sensitivity of 89.7% and a specificity of 99.3%. The cut-off value was 30% (Table 1, Additional file 1: Fig. S3) with the uncertain region (grey zone) ranging from 20 to 30%, which was considered as negative. Among the 1500 control sera were ten sera tested positive in the ELISA. When seven of the ten samples that were available in sufficient quantities were probed in an immunoblot against the N-protein, no bands were detected. As neither the N-protein nor any contamination, such as an *E. coli* protein, were recognized (Additional file 1: Fig. S4), we considered these samples as false positive in ELISA.

**Table 1:** Performance characteristics of the in-house SARS-CoV-2 N-protein based ELISA

	PCR	Symptom onset	PCR + symptom onset
*Sensitivity*			
[+] 0–6 d^a^	40.9% (20.7–63.7%)	0%	39.1% (19.7–61.5%)
[+] 7–13 d^b^	86.4% (65.1–97.1%)	–	86.4% (65.1–97.1%)
[+] 14–55 d^c^	100% (89.2–100%)	79.4% (62.1–91.3%)	89.7% (79.9–95.8%)
*Specificity*			
SARS–CoV–2 [–]^d^	–	–	99.3% (98.8–99.68%)

Confidence intervals (95%) are given in brackets. The median of days after symptom onset/PCR for the group [+] 14–55 d was 33 d

^a^Sample cohort consisted of 23 RT-PCR confirmed SARS-CoV-2 infections including one with documented symptom onset

^b^Sample cohort consisted of 22 RT-PCR confirmed SARS-CoV-2 infections

^c^Sample cohort consisted of 68 RT-PCR confirmed SARS-CoV-2 infections including 34 with documented symptom onsets

^d^Sample cohort consisted of 1500 control sera collected before 2015

As the N-protein was coated in ELISA using non-denaturing conditions but probed in the immunoblot after being denatured by SDS-PAGE, the seven false-positive samples were tested again in ELISA, but this time after coating the plate with denatured N-protein (Additional file 1: Fig. S5). Compared to the N-protein ELISA using native coating conditions, the absorbance decreased on average for positive samples by ~ 7% and ~ 65% for the false-positive and borderline samples (up to 90% for one false-positive sample). Thus, denaturing conditions identified 50% of the false-positive and 78% of borderline samples improving the specificity to 99.7%. An RNase/DNase digestion of purified N-protein prior to denaturation did not provide further improvement (data not shown).

### Validation of the N-protein ELISA

**Fig. 2:**
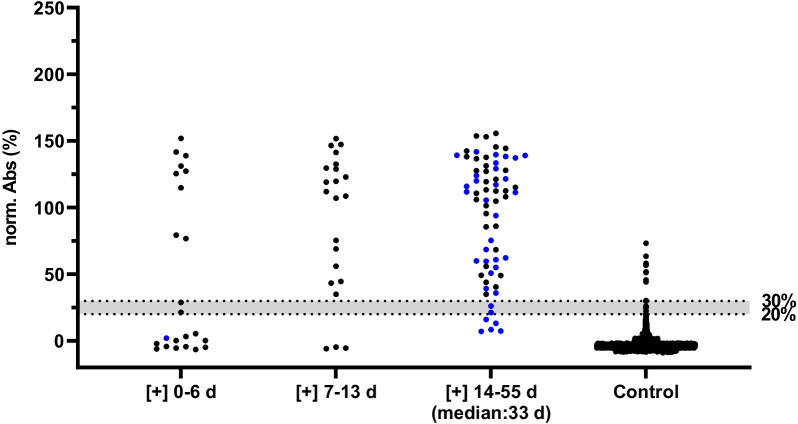
SARS-CoV-2 N-protein ELISA. In total 113 sera samples were collected from persons infected with SARS-CoV-2 and grouped based on the time period from symptom onset (blue) and for samples with unknown symptom onset by the date of a positive PCR test (black) until the blood sample was taken: 0–6 days (group 1, n = 23), 7–13 days (group 2, n = 22), and 14–55 days (group 3, n = 68, median: 33 d) as well as 1500 serum samples collected before 2015. Results were normalized by defining the positive control as 100% and negative control as 0% for each plate. The cut-off value was 30% and the grey zone below the cut-off ranging from 20 to 30% was considered as negative

Serum samples collected from persons infected with SARS-CoV-2 were assorted based on the time period passed since confirmation of the disease by a positive PCR result, i.e., sera collected within the first six days (group 1, n = 23), from day 7 to day 13 (group 2, n = 22), and on day 14–55 (group 3, n = 68). Our ELISA correctly identified nine sera of group 1 (39.1%), 19 sera of group 2 (86.4%), and 61 sera of group 3 (89.7%) as positive (Table 1, Fig. 2). The specificity of 99.3% was maintained with only 10 of 1500 negative samples being repeatedly recognized as positive (Table 1, Fig. 2). Patients with a history of COPD and smoking as well as gender and obesity had no significant effect on the false-positive rates (P > 0.05; Table 2).

**Table 2:** Diagnostic specificity of the in-house N-protein based ELISA

Sample group	Number	Negative	Borderline	Positive	Specificity (%)
SARS-CoV-2 [–]	1500	1482	8	10	99.3
COPD	276	273	0	3	98.9
Non-smokers	684	676	5	3	99.6
Light smokers	457	451	2	4	99.1
Heavy smokers	359	355	1	3	99.2
Male	710	699	4	7	99.0
Female	789	782	4	3	99.6
Normal weight	495	489	4	2	99.6
Pre-obesity	550	543	4	3	99.5
Obesity grade I	238	237	0	1	99.6
Obesity grade II	116	113	0	3	97.4
Obesity grade III	100	99	0	1	99.0

Subgroups are partially overlapping. Specificity was calculated as (“Negative” + “Borderline”)/“Total number”

**Fig. 3:**
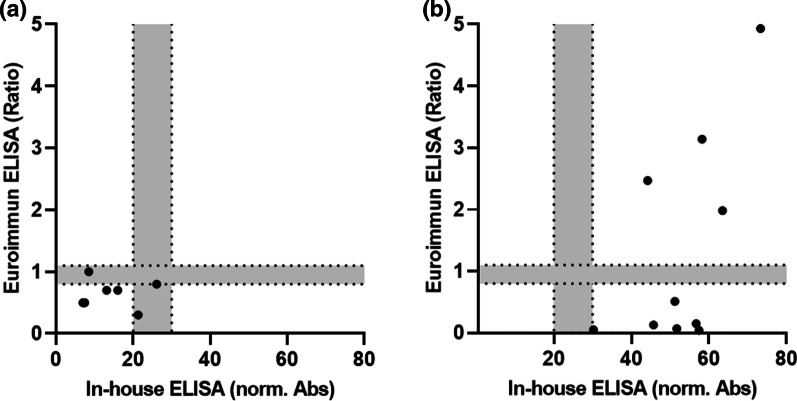
Serum samples incorrectly identified as negative and positive by the Euroimmun ELISA. **a** Among the 68 samples collected more than 14 days after symptom onset or a positive PCR test were seven samples missed (false-negative). **b** Ten samples detected as false-positive among 1500 samples collected before 2015 in our in-house N-protein ELISA (x-axis) were tested using the Euroimmun ELISA (y-axis). Borderline areas were 0.8–1.1 for the Euroimmun ELISA and 20–30% for the in-house ELISA, as indicated in grey

The seven serum samples from group 3 tested negative for IgG antibodies against the SARS-CoV-2 N-protein with the in-house ELISA were also negative when tested with the CE-labelled (European Economic Area) anti-SARS-CoV-2 NCP IgG ELISA (Euroimmun) (Fig. 3a). This confirms previous reports indicating that anti-N-protein IgG antibodies cannot be detected by ELISA in sera from some patients despite a PCR confirmed SARS-CoV-2 infection [9, 14].

**Fig. 4:**
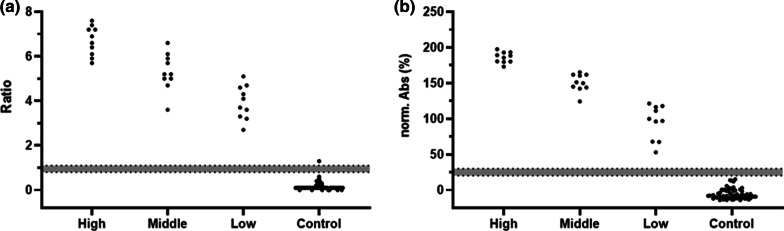
Comparison of Euroimmun (**a**) and in-house (**b**) N-protein based ELISA. Ten high, middle, and low positive sera collected 14 days after PCR confirmation and 33 random SARS-CoV-2 [-] serum samples were analyzed

When the ten serum samples tested false positive in our ELISA were tested with the CE-labelled anti-SARS-CoV-2 NCP IgG ELISA, four samples remained positive and six were negative (Fig. 3b). It should be noted that the ten samples tested here were selected from initially 1500 sera based on false positive results of our in-house assay. It was not our intention to determine the specificity of the commercial assay for all 1500 control samples, but we randomly selected 30 positive sera from group 3 tested with high, middle, and low OD_450_-values (10 sera per OD-range) in our ELISA and 33 of the 1490 remaining negative control serum samples. While the commercial ELISA confirmed all positive samples, one control sample negative in our in-house ELISA was detected as positive (Fig. 4). It should be noted that the resulting specificity of 96.9% for the commercial ELISA, which is well below the specificity of 99.8% reported by Euroimmun (Additional file 1: Table S6), should not be over-interpreted due to the low case numbers of the random sample set. Interestingly, both assays showed a similar trend in the calculated normalized absorbance and ratios from high to low readouts.

As the humoral immune system responds to a SARS-CoV-2 infection with IgA production before IgG is secreted [25], we tested also the 23 sera from group 1 and 29 randomly selected negative sera for IgA titers using the conditions of the optimized anti-N-protein ELISA but a secondary anti-IgA antibody. Only six sera were positive for IgA (26.1%) including four that were also positive for IgG (Additional file 1: Fig. S6). Considering that only two additional serum samples were correctly identified as positive, we did not further investigate an IgA-based ELISA.

### Interferences and cross reactivity

As hemoglobin, bilirubin, and triglycerides in serum and plasma samples may interfere with the ELISA, plasma samples were spiked with these substances at different concentrations. The OD_450_-values between the collected and the corresponding spiked plasma samples varied by less than 20% indicating that the in-house ELISA was not affected by these substances in the concentration range to be expected in human sera (Additional file 1: Fig. S7).

Finally, 26 serum samples with antibody titers confirmed for various other diseases, such as HIV 1/2, parvovirus B19, hepatitis A/B virus, cytomegalovirus, Epstein-Barr virus, and herpes simplex virus, and collected before 2019 were tested negative by the in-house N-protein based ELISA (Additional file 1: Fig. S8).

### Follow-up samples

For nine patients, follow-up samples were tested showing the seroconversion, increasing antibody titers during the second week after PCR confirmation, and relatively stable antibody titers for at least 54 days (Additional file 1: Fig. S9). Only the antibody titer of one patient decreased from day 9 but it remained positive for 65 days (Additional file 1: Fig. S9, light blue line).

## Discussion

Screening for SARS-CoV-2 infections is an important task for the prevention and treatment of COVID-19 and to control the ongoing pandemic. Direct detection methods, such as RT-PCR and antigen assays, are ideal for detecting viral RNA and viral proteins, respectively, as long as the virus replicates, but are limited by low detection rates afterwards [24]. Typically, the immune response starts with an early increase of specific IgM and IgA antibodies, but later, specific IgG levels increase and dominate already a few days after clinical symptoms started [11]. Thus, an accurate serological testing of anti-SARS-CoV-2 antibodies is important to understand the immune response, to detect previous infections, especially in people who cannot be vaccinated at the moment, such as children up to the age of 12 years or people refusing vaccinations, to control infections in immunized people and to validate an anticipated herd immunity.

Several serological assays have been reported, typically screening for IgG antibodies directed against the full-length spike (S-) protein, the outer S1 domain of the S-protein, or the receptor binding domain (RBD) [1, 22, 27]. Although the S-protein is considered as an important antigen of virus neutralizing antibodies and thus used in different vaccines, diagnostic assays relying on other proteins might be more sensitive and specific. A previous study validated two CE-labeled commercial S1-subunit and N-protein based ELISA assays with a moderate sensitivity of 91.8% and 84.8%, respectively [5]. Although the combination of both assays increased the sensitivity only to 93.2%, it indicates that S- and N-protein based ELISA are complementary to each other [5]. Moreover, a study on SARS-CoV-1 indicated that antibodies against the N-protein in serum persist longer than antibodies against the S-protein [17]. Therefore, a N-protein based serological test can improve the accuracy of antibodies detection for tracing viral infections for longer periods. Importantly, such assays will allow to differentiate between infected and non-infected vaccinated persons producing anti-S-protein antibodies (Additional file 1: Fig. S10), which will be increasingly important for epidemiological studies in the future.

Our in-house ELISA provided similar results as the CE-labeled commercial Euroimmun anti-SARS-CoV-2 NCP-ELISA (IgG), i.e., a ~ 90%-confirmation rate on the SARS-CoV-2-positive samples collected at least fourteen days after symptom onset or PCR diagnosis and a very good specificity of 99.3%. The specificity was further improved to 99.7% by testing positive samples in a second ELISA using the same N-protein, but denatured in 8 mol/L urea and coated in buffer containing 2 mol/L urea. This indicates that the ‘cross-reactivity’ is partially due to the presence of structural epitopes recognized by antibodies directed against other viral or bacterial proteins. However, this cross-reactivity is most likely not related to the infections with HIV 1/2, parvovirus B19, hepatitis A/B virus, cytomegalovirus, Epstein-Barr virus, and herpes simplex virus, as sera with confirmed infections and antibody titers these viruses were tested negative. Surprisingly, among the 23 sera of group 1, i.e., samples taken within the first 10 days after symptom onset or a positive PCR test, only six were positive for IgA (26.1%) compared to nine samples positive for IgG (39.1%). Thus, testing for IgA titers will most likely not significantly improve the sensitivity, but might reduce the specificity, which was not further investigated.

SARS-CoV-2 antibody tests are evolving at a rapid pace with more and more commercial test kits receiving a CE-label and The United States Food and Drug Administration (FDA) Emergency Use Authorization (EUA). However, the reported performances indicated that many are not suitable for the clinical practice. A recent study relying on one cohort of recovered patients previously infected with SARS-CoV-2 (363 sera), reported for five commercialized IgG antibody tests good specificities above 99.0% [6]. However, the sensitivities varied for two N-protein-based ELISA from 53.7% (Abbott) to 93.1% (Roche) and for two S1-protein-based ELISA from 77.1% (Euroimmun) to 89.2% (Immundiagnostic), which was not improved for a combined S1- and S2-protein based providing a sensitivity of 81.3% (DiaSorin). Compared to these tests, our in-house N-protein based ELISA provides a high sensitivity (89.7%) for samples collected at least 14 days after symptom onset or a positive PCR test and a high specificity of 99.3%, which was even improved to 99.7% when combined with the denatured N-protein ELISA. The assay sensitivity was mostly reduced by sera collected from patients with very mild symptoms, most likely as there was only weak response of the adaptive immune system. It should be noted that the sample sets tested here was different from the sample set used by Eberhardt et al. [6]. Thus, the calculated sensitivity and specificity cannot be directly compared.

## Conclusion

The in-house N-protein-based ELISA reported here provides a high diagnostic sensitivity of 89.7% in serum samples collected at least 14 days after symptom onset and PCR confirmation and a high specificity of 99.3%, after retesting denaturing conditions even 99.7%, allowing a reliable and robust serological testing of past SARS-CoV-2 infections. The protocol is fast (less than two hours), if precoated microtiter plates are used, and requires less than two microliters of serum. Thus, this assay should improve the screening for SARS-CoV-2 infections.

## Supplementary Information

**Additional file 1**. Clinical parameters and ELISA validation.

## Data Availability

All data generated or analyzed during this study are included in this published article and its supplementary information files.
