# Supplementary material for: Sensitive and specific serological ELISA for the detection of SARS-CoV-2 infections
Source: Virol J. 2022 Mar 19;19:50. doi: 10.1186/s12985-022-01768-4 (PMC8934124; doi:10.1186/s12985-022-01768-4)
Supplement: Supplementary file 1 — Additional file 1. Clinical parameters and ELISA validation. [file 12985_2022_1768_MOESM1_ESM.docx]

**Additional file**

**Sensitive and specific serological ELISA for the detection of SARS-CoV-2 infections**

Ji Luo^1,2,3^, Alexandra Brakel^1,2^, Andor Krizsan^1,2^, Tobias Ludwig^1,2^, Marina Mötzing^1,2^, Daniela Volke^1,2^, Nicole Lakowa^5^, Thomas Grünewald^5^, Claudia Lehmann^8^, Johannes Wolf^4^, Stephan Borte^4^, Sanja Milkovska-Stamenova^1,2,3^, Felix Fingas^3^, Markus Scholz^6,7^, and Ralf Hoffmann^1,2^

**Table of Contents**

Additional file 1: Table S1: Clinical parameters of patient samples S2

Additional file 1: Table S2: Clinical parameters of Krankenhaus Nordwest S3-S5

Additional file 1: Table S3: Clinical parameters of Klinikum Chemnitz gGmbH S6

Additional file 1: Table S4: Clinical parameters of Klinikum St Georg gGmbH S7-S8

Additional file 1: Table S5: Clinical parameters of negative control samples S9

Additional file 1: Table S6: Performance of the Euroimmun IgG ELISA for SARS-CoV-2 S10

Additional file 1: Figure S1: Storage stability of in-house N-protein S11

Additional file 1: Figure S2: Optimization of the Nucleocapsid-protein ELISA S12

Additional file 1: Figure S3: Receiver Operating Characteristics curves S13

Additional file 1: Figure S4: Immunoblot of false positive samples S14

Additional file 1: Figure S5: Denatured protein test S15

Additional file 1: Figure S6: Interference S16

Additional file 1: Figure S7: Cross-reactivity S17

Additional file 1: Figure S8: Time-course antibody titer S18

Additional file 1: Figure S9: IgA-ELISA S19

Additional file 1: Figure S10: N-Protein ELISA of vaccinated people S19

Additional file 1: Table S1. Clinical parameters of 113 serum samples obtained from patients with confirmed SARS-CoV-2 infections (Part of gender information was missing). Samples were grouped based on the days passed after symptom onset or, if this was unknown, days passed since a positive PCR test. For detailed information about the different sample sets see tables S2 to S4.

| Variables | [+] <7 d | [+] 7-14 d | [+] >14 d |
| --- | --- | --- | --- |
| Total number | 23 | 22 | 68 |
| Sex (M/F) | 8/6 | 1/3 | 26/27 |
| Age, median (range) | 77 (19-97) | 68 (42-97) | 74 (24-97) |

Additional file 1: Table S2: Clinical parameters of 82 serum samples obtained from 32 patients aged between 42 and 97 (mean age of 69 years) with confirmed SARS-CoV-2 infections and hospitalized at Krankenhaus Nordwest in Frankfurt in the period from March 27^th^ to June 2^nd^ 2020. Samples were collected 0 to 65 days after PCR (mean of 23 days)

| **Patient** | **age** | **Sample** | **Day after PCR** |
| --- | --- | --- | --- |
| P1 | 55 | F1 | 9 |
| P2 | 60 | F2 | 10 |
| P3 | 97 | F3 | 4 |
|  |  | F4 | 15 |
|  |  | F5 | 11 |
| P4 | 75 | F6 | 17 |
| P5 | n/a | F7 | 8 |
|  |  | F8 | 29 |
|  |  | F9 | 16 |
| P6 | 80 | F10 | 15 |
|  |  | F11 | 7 |
| P7 | 81 | F12 | 25 |
|  |  | F13 | 33 |
|  |  | F14 | 40 |
|  |  | F15 | 42 |
|  |  | F16 | 54 |
|  |  | F17 | 17 |
| P8 | 76 | F18 | 21 |
|  |  | F19 | 29 |
|  |  | F20 | 36 |
|  |  | F21 | 43 |
|  |  | F22 | 45 |
|  |  | F23 | 9 |
| P9 | 69 | F24 | 9 |
| P10 | 80 | F25 | 18 |
|  |  | F26 | 7 |
| P11 | 59 | F27 | 25 |
|  |  | F28 | 34 |
|  |  | F29 | 39 |
|  |  | F30 | 45 |
|  |  | F31 | 46 |
|  |  | F32 | 16 |
| P12 | n/a | F33 | 24 |
|  |  | F34 | 36 |
|  |  | F35 | 7 |
|  |  | F36 | 14 |
| P13 | 89 | F37 | 5 |

| **Patient** | **age** | **Sample** | **Day after PCR** |
| --- | --- | --- | --- |
| P14 | 83 | F38 | 23 |
|  |  | F39 | 30 |
|  |  | F40 | 39 |
|  |  | F41 | 10 |
| P15 | 66 | F42 | n/a |
| P16 | 56 | F43 | 40 |
|  |  | F44 | 14 |
| P17 | 62 | F45 | 2 |
|  |  | F46 | 26 |
|  |  | F47 | 33 |
|  |  | F48 | 42 |
|  |  | F49 | 48 |
|  |  | F50 | 53 |
|  |  | F51 | 13 |
| P18 | 79 | F52 | 47 |
|  |  | F53 | 19 |
|  |  | F54 | 12 |
| P19 | 86 | F55 | 18 |
| P20 | 42 | F56 | 22 |
|  |  | F57 | 26 |
|  |  | F58 | 38 |
|  |  | F59 | 47 |
|  |  | F60 | 47 |
|  |  | F61 | 58 |
|  |  | F62 | 65 |
|  |  | F63 | 9 |
| P21 | 65 | F64 | 53 |
| P22 | 81 | F65 | 5 |
| P23 | 53 | F66 | 16 |
| P24 | 79 | F67 | 3 |
|  |  | F68 | 15 |
| P25 | 57 | F69 | 0 |
|  |  | F70 | 5 |
| P26 | 62 | F71 | 2 |
|  |  | F72 | 7 |
|  |  | F73 | 9 |
| P27 | 54 | F74 | 5 |
| P28 | 48 | F75 | 7 |
|  |  | F76 | 9 |
| P29 | 85 | F77 | 13 |
| P30 | 80 | F78 | n/a |
|  |  | F79 | n/a |
|  |  | F80 | n/a |
| **Patient** | **age** | **Sample** | **Day after PCR** |
| P31 | 58 | F81 | n/a |
| P32 | 54 | F82 | 8 |

Additional file 1: Table S3: Clinical parameters of 32 serum samples obtained from patients with confirmed SARS-CoV-2 infections and hospitalized at the Klinikum Chemnitz gGmbH in the period from March 21^st^ to April 15^th^ 2020. Seventeen males and fifteen females aged 26 to 88 years (mean age of 67 years) were included in the study. One sample of each patient was collected 0 to 41 days after PCR (mean of 17 days).

| **Sample** | **Sex** | **Age** | **Days after PCR** | **NIAID-OS score** |
| --- | --- | --- | --- | --- |
| C1 | female | 67 | 0 | 5 |
| C2 | female | 62 | 6 | 5 |
| C3 | male | 68 | 1 | 5 |
| C4 | male | 78 | 0 | 8 |
| C5 | female | 88 | 5 | 5 |
| C6 | male | 76 | 2 | 5 |
| C7 | male | 82 | 5 | 4 |
| C8 | female | 79 | 1 | 5 |
| C9 | male | 80 | 3 | 5 |
| C10 | male | 37 | 2 | 4 |
| C11 | male | 75 | 4 | 5 |
| C12 | female | 75 | 0 | 5 |
| C13 | female | 67 | 10 | 5 |
| C14 | male | 66 | 9 | 5 |
| C15 | female | 75 | 14 | 5 |
| C16 | female | 67 | 33 | 5 |
| C17 | male | 68 | 23 | 5 |
| C18 | male | 80 | 36 | 5 |
| C19 | male | 57 | 41 | 5 |
| C20 | female | 51 | 24 | 2 |
| C21 | male | 67 | 25 | 4 |
| C22 | female | 64 | 25 | 5 |
| C23 | male | 58 | 32 | 5 |
| C24 | female | 33 | 33 | 4 |
| C25 | female | 54 | 35 | 5 |
| C26 | male | 56 | 32 | 5 |
| C27 | female | 26 | 31 | 4 |
| C28 | male | 66 | 27 | 5 |
| C29 | male | 82 | 18 | 4 |
| C30 | female | 79 | 20 | 5 |
| C31 | male | 81 | 16 | 5 |
| C32 | female | 71 | 20 | 5 |

Additional file 1: Table S4: Clinical parameters of 43 serum samples obtained from patients with confirmed SARS-CoV-2 infections and hospitalized at the Klinikum St. Georg gGmbH in the period from April 2^nd^ to April 26^th^ 2020. Eighteen males and 21 females aged between 15 and 90 (mean age of 49 years) were included in the study. One sample of each patient was collected 23 to 51 days after PCR (mean of 30 days) and 23 to 55 days after symptom onset (mean of 40 days).

| **Sample** | **Sex** | **Age** | **Days after symptom onset** | **Days after PCR** | **WHO score** |
| --- | --- | --- | --- | --- | --- |
| SG1 | female | 34 | 35 | 29 | 2 |
| SG2 | female | 41 | 38 | 29 | 2 |
| SG3 | male | 38 | 34 | 33 | 2 |
| SG4 | female | 64 | 33 | 0 | 4 |
| SG5 | female | 54 | 43 | 42 | 2 |
| SG6 | female | 64 | 37 | 34 | 2 |
| SG7 | male | 78 | 40 | 44 | 3 |
| SG8 | female | 54 | 47 | 44 | 2 |
| SG9 | female | 52 | 35 | 0 | 4 |
| SG10 | male | 52 | 55 | n/a | 3 |
| SG11 | male | 50 | 48 | 44 | 2 |
| SG12 | female | 39 | 38 | 33 | 2 |
| SG13 | female | 90 | 34 | 31 | 4 |
| SG14 | male | 47 | 44 | n/a | 3 |
| SG15 | female | 58 | 35 | 0 | 4 |
| SG16 | male | 55 | 36 | 1 | 4 |
| SG17 | male | 59 | 38 | 0 | 4 |
| SG18 | male | 54 | 44 | 42 | 2 |
| SG19 | female | 65 | 33 | 32 | 4 |
| SG20 | female | 39 | 34 | 31 | 2 |
| SG21 | male | 24 | 23 | 51 | 2 |
| SG22 | female | 64 | 53 | 44 | 4 |
| SG23 | female | 39 | 38 | 33 | 2 |
| SG24 | male | 63 | 51 | 30 | 2 |
| SG25 | male | 63 | 45 | 30 | 4 |
| SG26 | male | 49 | 34 | 32 | 2 |
| SG27 | female | 54 | 42 | 36 | 2 |
| SG28 | male | 42 | 34 | 32 | 2 |
| SG29 | female | 40 | 34 | 29 | 2 |
| SG30 | male | 35 | 38 | 33 | 2 |
| SG31 | male | 25 | 3 | n/a | 3 |
| SG32 | female | 38 | 54 | 52 | 2 |
| SG33 | female | 37 | n/a | n/a | 2 |
| SG34 | female | 51 | 31 | 31 | 2 |
| SG35 | male | 53 | 49 | 55 | 2 |
| SG36 | female | 45 | n/a | 48 | 2 |
| SG37 | male | 28 | 44 | 39 | 2 |
| SG38 | male | 42 | 55 | 46 | n/a |
| SG39 | female | 80 | n/a | 0 | 4 |
| SG40 | male | 17 | 45 | 33 | 2 |
| SG41 | male | 15 | 38 | 0 | n/a |

Additional file 1: Table S5. Clinical parameters of 1,500 control serum samples collected from 2009 to 2014 (SARS-CoV-2 negative) and the corresponding specificity of the in-house ELISA using the N-protein expressed and purified in-house.

| Diagnostic parameter | Value | Specificity of in-house ELISA |
| --- | --- | --- |
| Gender | Male (47.4%)  Female (52.6%) | 99.0%  99.6% |
| Age (years) | 20-39 (5.1%)  40-49 (30.0%)  50-59 (24. 5%)  60-69 (23.7%)  >70 (16.7%) | 100.0%  99.6%  99.7%  99.2%  98.4% |
| BMI | 18.5 – 25: Normal weight (32.3%)  25 – 30: Pre-Adipositas (36.7%)  30 – 35: Adipositas grade I (15.9%)  35 – 40: Adipositas grade II (7.7%)  > 40: Adipositas grade III (6.7%) | 99.6%  99.5%  99.6%  97.4%  99.0% |
| COPD | Yes (18.5%)  No (81.5%) | 98.9%  99.3% |

Additional file 1: Table S6. Performance characteristics of the Euroimmun IgG ELISA kit for SARS-CoV-2 positive samples and negative serum samples, as reported by the manufacturer (Euroimmun flyer EI_2606-2G_A_DE_C01.doc from April 30^th^, 2020).

| **Sample** | **Sensitivity** | **Specificity** | **Number of samples** |
| --- | --- | --- | --- |
| Symptom onset or PCR [+] ≤ 9 d | 80.0% | - | 15 |
| Symptom onset or PCR [+] ≥ 10 d | 94.6% | - | 56 |
| SARS-CoV-2 [-] | - | 99.8% | 1248 |

Additional file 1: Figure S1. Storage stability of in-house N-protein for 360 days. N-Protein was stored at 4 °C and -20 °C in PBS + 200 mM NaCl with and without 10% or 25% of Glycerol and additionally at -80 °C only in PBS + 200 mM NaCl. Protein concentrations of N-Protein aliquots stored at different conditions over the time period of 360 days (A). SDS-PAGE analysis of 1, 2 and 4 µg loaded N-Protein from aliquots stored in PBS + 200 mM NaCl at different temperatures (B) and from aliquots stored in PBS + 200 mM NaCl with 10% or 25% of Glycerol at 4 °C and -20 °C for 1, 180 and 360 days (C).


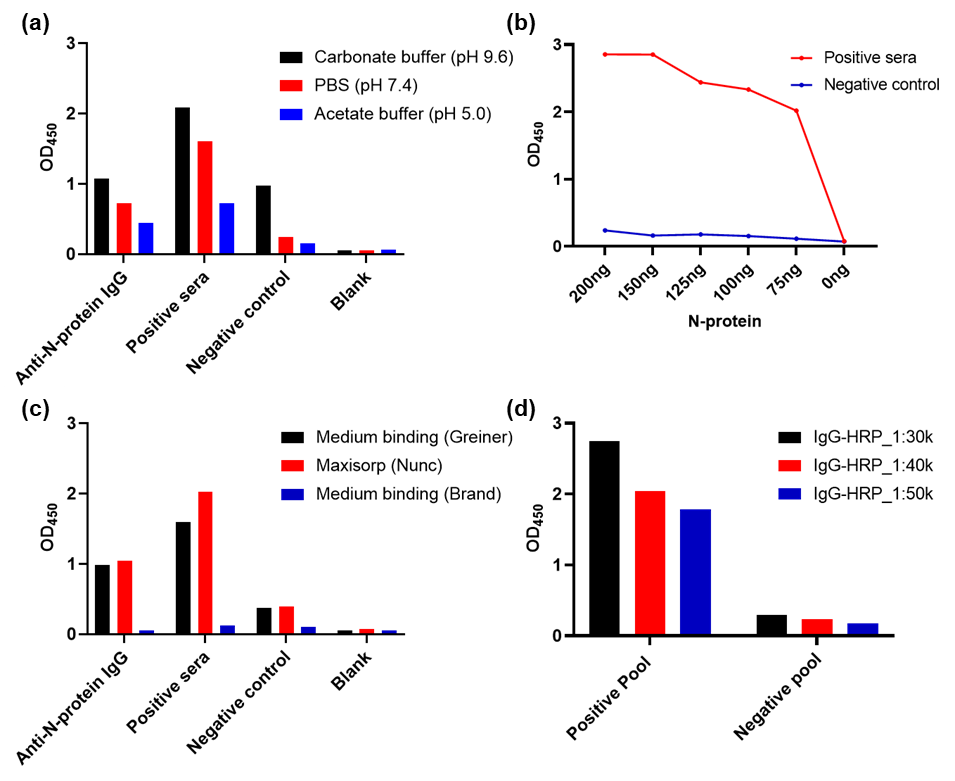


Additional file 1: Figure S2. Optimization of the N-protein ELISA by testing different (a) coating buffers, (b) N-protein quantities, (c) plate types, and (d) dilutions of the secondary anti-IgG antibody.


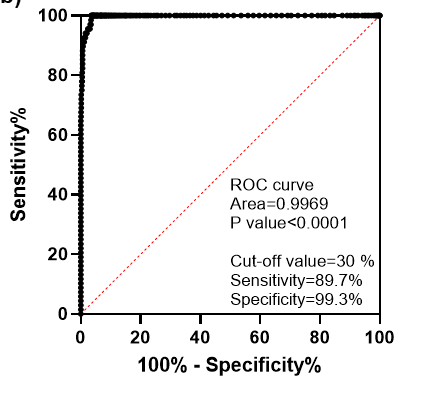


Additional file 1: Figure S3. Receiver operating characteristics (ROC) curve for the purified N-protein in-house ELISA considering data from 68 serum samples collected at least 14 days after symptom onset or PCR confirmation of a SARS-CoV-2 infection and 1500 negative serum samples collected in the years 2009 to 2014.

Additional file 1: Figure S4. Immunoblots of commercial and in-house N-protein probed with one positive, one negative, and seven sera of the control group that appeared positive in the N-protein ELISA. The N-protein quantities loaded on the gel were 0.5 µg. M: marker proteins with the indicated molecular masses. N: N-protein. Only the SARS-CoV-2 positive serum but none of the sera considered as false positive detected the purified in-house N-protein (green bands). The commercial N-protein was recognized by sera 1 and 5.


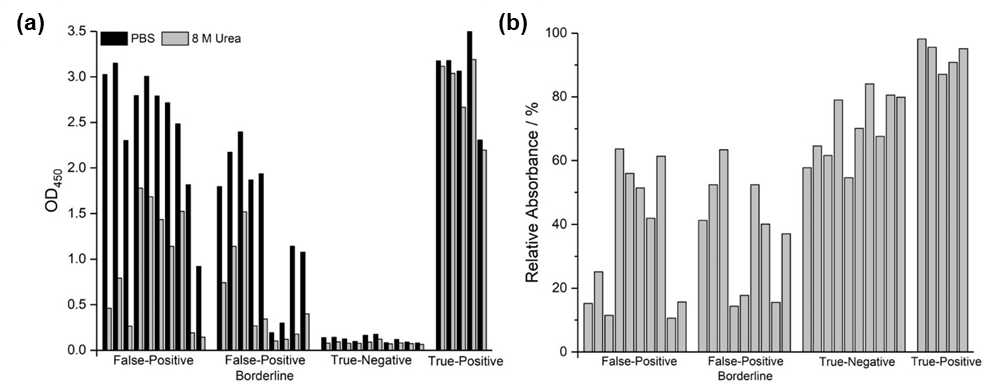


Additional file 1: Figure S5**:** Re-examination of ten false-positive and nine borderline samples using native and denaturing conditions to coat the N-protein. The N-protein (150 ng/well) was either dissolved in PBS at room temperature or in PBS containing 8 mol/L urea followed by an incubation at 37 °C (1 h) before it was coated overnight (4 °C). For control, ten true-negative and five true-positive samples were tested. The (a) OD_450_ values recorded for denaturing conditions were (b) normalized to OD_450_ of the corresponding sample tested with native conditions (= 100%).


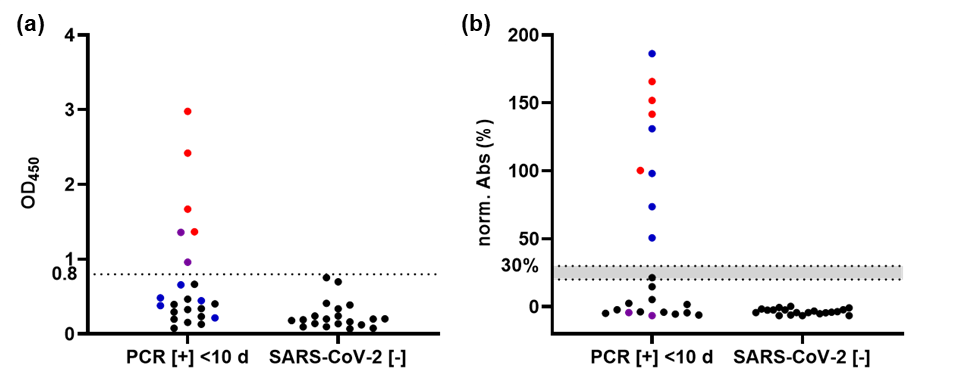


Additional file 1: Figure S6. In-house N-protein ELISA using secondary (a) anti-IgA and (b) IgG antibodies to test SARS-CoV-2 samples of group 1 (n = 23) and negative control samples (n = 29). Samples tested positive in both ELISA are colored red, while purple and blue symbols indicate samples tested positive only for IgA and IgG, respectively.

Additional file 1: Figure S7. Influence of interfering substances: hemoglobin (up to 20 g/L), bilirubin (up to 0.3 g/L), and triglycerides (up to 15 g/L) on the absorbance of an antibody negative (black) and a positive sample (red). Each sample was tested three times in parallel and at least twice on different days. Dotted lines indicate average absorbance of the original plasma and the grey zone indicated a deviation of ±20%.

Additional file 1: Figure S8. Normalized absorbances obtained by the in-house ELISA for serum samples tested positive for HIV 1/2, parvovirus B19, hepatitis A/B virus, cytomegalovirus, Epstein-Barr virus, and herpes simplex virus. The grey zone indicates the range of 10% below the cut-off, which was considered negative.


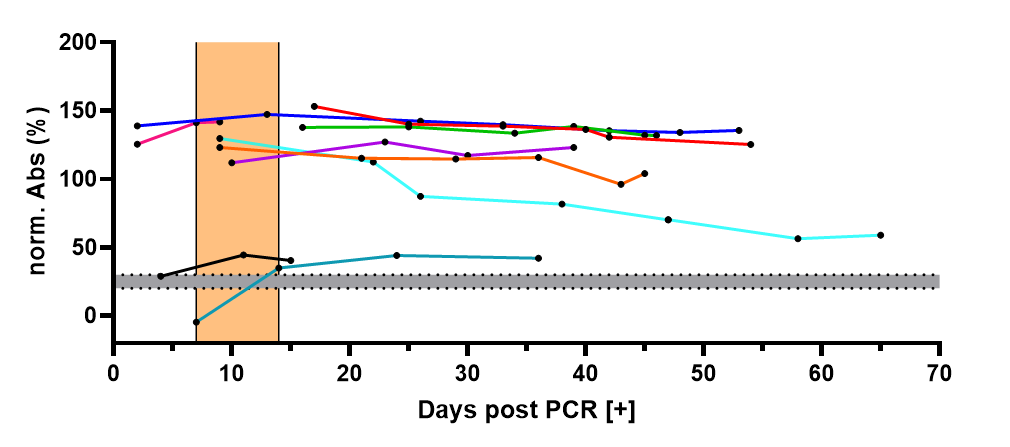


Additional file 1: Figure S9. Time course of antibody titers of nine individual patients from Kankenhaus Nordwest. The second week after PCR diagnosis is highlighted in orange. The cut-off value was 30%, the grey zone shows the range of 10% below the cut-off.

Additional file 1: Figure S10. Normalized absorbances obtained by the in-house N-protein ELISA (a) and an in-house RBD ELISA (b) using serum samples of non-vaccinated (n=7), vaccinated people without SARS-CoV-2 infection (n=10), and people that were infected after the second vaccination (n=7). Serum samples of were taken one week after the second vaccination. Serum samples of vaccinated and subsequently infected people were taken 13 to 67 days (median: 22 days) after RT-PCR confirmed SARS-CoV-2 infection. Borderline area was 20% to 30% for the in-house N-protein ELISA, as indicated in grey.
